# Amplitude, Latency, and Peak Velocity in Accommodation and Disaccommodation Dynamics

**DOI:** 10.1155/2017/2735969

**Published:** 2017-10-31

**Authors:** Antonio J. Del Águila-Carrasco, José J. Esteve-Taboada, Eleni Papadatou, Teresa Ferrer-Blasco, Robert Montés-Micó

**Affiliations:** Department of Optics and Optometry and Vision Sciences, University of Valencia, Calle Dr. Moliner, 50, Burjassot, 46100 Valencia, Spain

## Abstract

The aim of this work was to ascertain whether there are differences in amplitude, latency, and peak velocity of accommodation and disaccommodation responses when different analysis strategies are used to compute them, such as fitting different functions to the responses or for smoothing them prior to computing the parameters. Accommodation and disaccommodation responses from four subjects to pulse changes in demand were recorded by means of aberrometry. Three different strategies were followed to analyze such responses: fitting an exponential function to the experimental data; fitting a Boltzmann sigmoid function to the data; and smoothing the data. Amplitude, latency, and peak velocity of the responses were extracted. Significant differences were found between the peak velocity in accommodation computed by fitting an exponential function and smoothing the experimental data (mean difference 2.36 D/s). Regarding disaccommodation, significant differences were found between latency and peak velocity, calculated with the two same strategies (mean difference of 0.15 s and −3.56 D/s, resp.). The strategy used to analyze accommodation and disaccommodation responses seems to affect the parameters that describe accommodation and disaccommodation dynamics. These results highlight the importance of choosing the most adequate analysis strategy in each individual to obtain the parameters that characterize accommodation and disaccommodation dynamics.

## 1. Introduction

Ocular accommodation is the ability of the eye to focus on objects that are located at different distances [1]. A change in focus from far onto a near object is referred to as accommodation, and it means an increase in the optical power of the eye. Contrarily, a change in focus from near onto a far object is known as disaccommodation (relaxation of accommodation), and it means a decrease in the optical power of the eye. Accommodation dynamics have been extensively studied [2–9]. Two typical accommodation demand patterns have been used for this purpose in the past: sinusoidal [10–14] and pulse changes [7, 15–17]. However, sinusoidal changes in demand are not suitable to properly evaluate first- or second-order dynamics of accommodation, that is, velocity and acceleration. To study these aspects of accommodation, pulse changes in demand have been typically used, from which velocity and acceleration profiles can be calculated, and different parameters about the first- and second-order dynamics of accommodation can be extracted, for example, peak velocity and acceleration, time to peak velocity, and time to peak acceleration.

It is well known how accommodation dynamics vary with age [7, 8], refractive state [18–20], or even the starting accommodation demand [21] or amplitude of the step-change [6, 21]. Regarding the analysis of pulse changes in accommodation demand, different studies have used different methodologies to characterize the accommodation response to pulse changes in demand. There are studies where exponential functions were fitted to the experimental responses [7, 21] and others where a sigmoid function was preferred over the exponential [22, 23], and in some studies the response was directly analyzed or was fitted to other functions [9, 16]. Even after selecting one particular methodology, there are different strategies to find, for instance, the start and the end of the response. The values obtained in different studies for the different parameters are very subject-dependent and, at times, contradictory. Thus, it would be interesting to know whether the methodology chosen to compute them has any effect on the outcome.

Three parameters regarding accommodation and disaccommodation dynamics were considered in this study: amplitude, latency, and peak velocity of the responses. These are of the most studied and well-known parameters in accommodation dynamics [7, 8].

The aim of this work was then to elucidate whether the use of different analysis strategies when characterizing accommodation and disaccommodation dynamics can yield different results in representative parameters, in particular, the amplitude, latency, and peak velocity of the responses.

## 2. Materials and Methods

### 2.1. Participants

Four subjects were enrolled to participate in this study. The participants had an average (±SD) age of 28 (±2) years and a mean spherical refractive error of −0.19 (±0.55) D. None of the participants exhibited astigmatism greater than 1 D. Subjects presented no ocular pathologies and no accommodation anomalies. The study adhered to the tenets of the Declaration of Helsinki and informed consent was obtained from all the subjects after explanation of the nature and possible consequences.

### 2.2. Apparatus

An adaptive-optics system was used to measure the accommodation response of the subjects. This system is composed of a 1024 microlenses Shack-Hartmann aberrometer, a Mirao-52e (Imagine Eyes, France) deformable mirror, an 800 × 600 pixels microdisplay, and a motorized Badal system. The target was a black Maltese cross on a white background, spanning 1.25° of visual angle and with a luminance at the corneal plane of about 25 cd/m^2^. The target was seen through a circular artificial pupil of 4 mm in diameter.

All measurements were taken using custom-made software in MATLAB (MathWorks, Inc., Natick, MA, USA), based on the analysis and simulation software library and software development kits provided by the manufacturer (Imagine Eyes).

### 2.3. Experimental Procedure

The right eye of each subject was measured, while the contralateral eye was occluded. Subjects were properly aligned with the device with the help of a chin and forehead support. Subjects' pupil was monitored in real time with an infrared camera so to avoid displacements while measuring.

Before measuring the accommodation responses, the far point of the subjects was determined with a fogging methodology [24] by moving the Badal system. First, the target was moved far away from the subjects' far point until they saw it blurred. Then, the target was moved closer to the eye in 0.25 D steps until it first became clear. The use of this strategy avoided unintentional use of accommodation.

After the far point was determined, the accommodation response of the subjects to a step-change in accommodation demand was measured at 20 Hz with the Shack-Hartmann aberrometer during trials lasting 5 s. The deformable mirror introduced rapidly the pulse-change in demand. The pulse had 2 D of amplitude and occurred randomly between 0.5 and 1 s after the trial started. Both accommodation and disaccommodation were recorded. For the accommodation assessment, the demand changed rapidly from 0 to 2 D, whereas for the disaccommodation, the demand changed rapidly from 2 to 0 D. Three trials were presented to each subject for accommodation and disaccommodation.

### 2.4. Data Analysis

**Figure 1:**
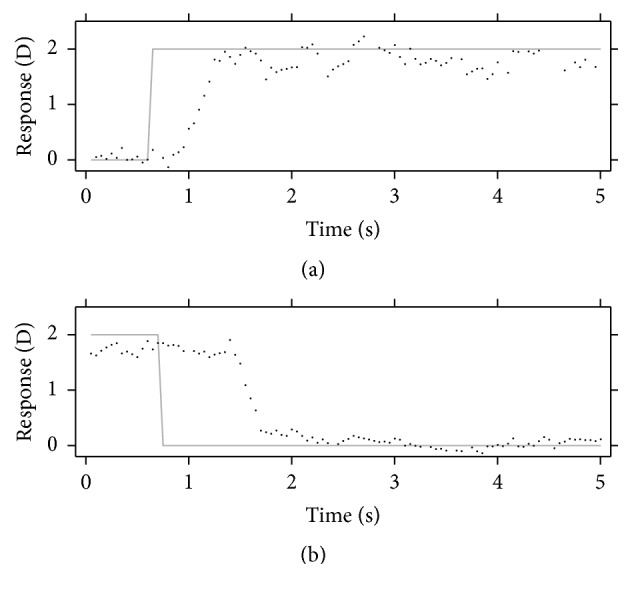
Example of accommodation (a) and disaccommodation (b) responses. Black points show experimental data and gray solid lines represent the accommodation demand.

Accommodation and disaccommodation responses were calculated by means of the least-square fitting method, using the Zernike spherical defocus term *C*_2_^0^, as previously described [25]. From these responses, several parameters were extracted: peak velocity, latency, and accommodation amplitude. Three different methods were used for determining these parameters.  Figure 1 shows a typical example of accommodation and disaccommodation responses obtained in this study.

**Figure 2:**
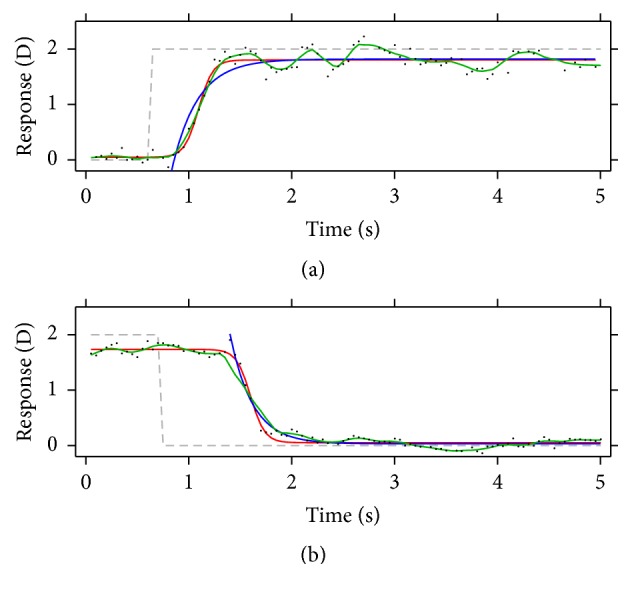
Different function fitted to the experimental data for accommodation (a) and disaccommodation (b) responses. Blue curves show the best exponential fit to the data; red curves show the best sigmoid fit to the data; green curves show smoothed responses. Black points represent the experimental data and gray dashed lines represent the accommodation demand, as in Figure 1.

**Figure 3:**
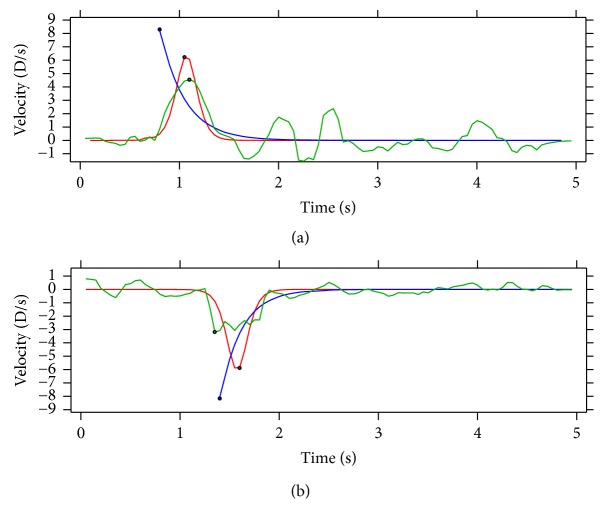
Accommodation (a) and disaccommodation (b) velocity plots. Blue curves show the velocity obtained when an exponential function was fitted to the experimental data; red curves show the velocity obtained when a Boltzmann sigmoid function was fitted to the experimental data; green curves show the velocity obtained when experimental data were smoothed. The peak velocity obtained for each method is indicated with markers.

The first method consisted of fitting the experimental data to the following exponential function (see blue curves in Figure 2)

(1) $$\begin{array}{c} r=r_{0}\pm a\left(\;1-e^{-t/\tau }\;\right), \end{array}$$

where *r* stands for the accommodation response at each moment in D, *t* is the time in seconds, *r*_0_ represents the initial value of the response in D, *a* represents the amplitude of the response in D, and *τ* represents the time constant. This is the function fitted to the accommodation responses; for the disaccommodation, the plus sign becomes negative. There is some latency between the instant the accommodation demand changes and the instant the eye starts responding. This latency needs to be removed before fitting the exponential function to the experimental data. To account for this latency, a previously utilized algorithm was applied [6, 26]. Basically, as described in [6], “the algorithm searched for three consecutive increasing data values, followed by four consecutive data values in which no two consecutive decreases occurred. When these criteria were met, the first data point in the sequence was recorded as the start of the response.” The exponential function with minus sign and the inverse algorithm were used for disaccommodation. The peak velocity (see blue markers in Figure 3) was extracted by solving the first derivative of the exponential function when *t* = 0, resulting in

(2) $$\begin{array}{c} peakVel=\pm \frac{a}{\tau }, \end{array}$$

where the plus sign refers to accommodation and the minus sign to disaccommodation.

The second method consisted of fitting the experimental data to a Boltzmann sigmoidal function (see red curves in Figure 2), defined by the equation

(3) $$\begin{array}{c} r=\frac{r_{0}-r_{f}}{1+e^{\left(t-c\right)/s}}+r_{f}, \end{array}$$

where *r* is the accommodation response at each moment in D, *t* is the time in seconds, and *r*_0_ stands for the initial response and *r*_*f*_ for the final one, both of them in D. The parameter *c* indicates the time at which the sigmoid function reaches 50% of the total change, and it is given in seconds. The parameter *s* is related to the slope of the change. This type of fit has been used before with similar accommodation responses [22, 23]. From here, the accommodation amplitude was calculated as the difference between the final response and the initial one; the latency was calculated as the difference in time between the instant the accommodation demand changes and the instant when the response reaches 5% of the final response *r*_*f*_; and the peak velocity (see red markers in Figure 3) was calculated as the maximum or the minimum of the sigmoid derivative, depending on whether it was accommodation or disaccommodation, respectively.

In the last method, a robust version of a nonparametric local regression method,* lowess* [27], that assigns lower weight to outliers in the regression was used to smooth the experimental responses (see green curves in Figure 2), with the smoothing parameter set to 0.075. This smoothing parameter was chosen because it allows for a slight smoothing of the response, without losing too much information. From these smoothed responses, the latency was calculated as the difference in time between the instant the accommodation demand changes and the first sample point where the velocity of the response was greater than 0.5 D/s and continued to do so for the next 100 ms [16]. The peak velocity (see green markers in Figure 3) was calculated by finding the maximum (or minimum if it was disaccommodation) of the first derivative of the smoothed response. To avoid noise, a rectangular window was applied to the velocity, where values were set to zero before the accommodation demand changed. The response amplitude was calculated as the difference between the response obtained when the accommodation velocity fell below 90% of the peak velocity and continued to do so for the next 100 ms [16] and the response obtained just after the latency.

After confirming that all assumptions required were fulfilled, one-way ANOVAs were performed to the amplitude, latency, and peak velocity values to evaluate whether there were any differences among methodologies. The significance level was set at 0.05.

## 3. Results

**Figure 4:**
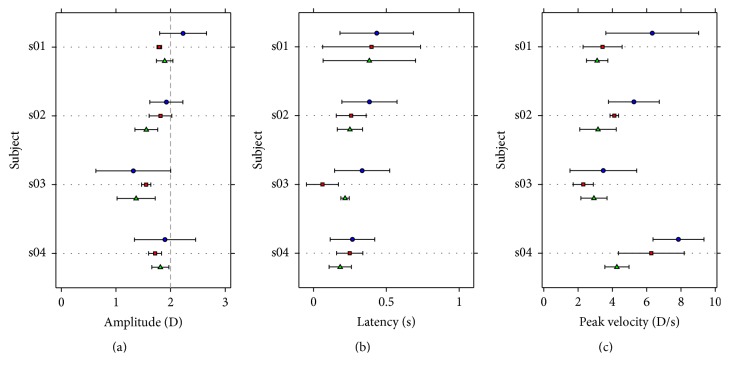
Mean obtained over the three trials for each subject of the accommodation parameters analyzed. (a) shows the mean amplitude of the accommodation response in diopters, (b) shows mean latency in seconds, and (c) shows peak velocity in diopters per second. Blue circles represent the mean obtained using the exponential fitting, red squares represent the mean obtained using the sigmoid fitting, and green triangles represent the mean obtained using the smoothing method. Error bars are ± standard deviation.

Figure 4 shows the average amplitude, latency, and peak velocity for each subject who took part in this study, computed using the three different methods explained before.

The values obtained for latency and peak velocity with the exponential fitting are systematically larger than those obtained using the other two methods. The amplitude of the response is also greater with the exponential fitting method, except for one subject.

The one-way ANOVA revealed that there were no statistically significant differences among methods for the response amplitude or the latency (*p* = 0.417 and *p* = 0.282, resp.). However, there were statistically significant differences in the peak velocity depending on the method used to compute it (*p* = 0.008). Post hoc pairwise comparisons by means of the Tukey-Kramer methods revealed statistically significant differences between the peak velocity calculated with the exponential fitting and the one calculated after smoothing the responses (*p* = 0.008). All the pairwise comparisons can be found in Table 1, where the mean of the differences between pairs of methods, together with the confidence interval limits, and the *p* value of post hoc multiple comparisons by means of the Tukey-Kramer method are shown.

**Figure 5:**
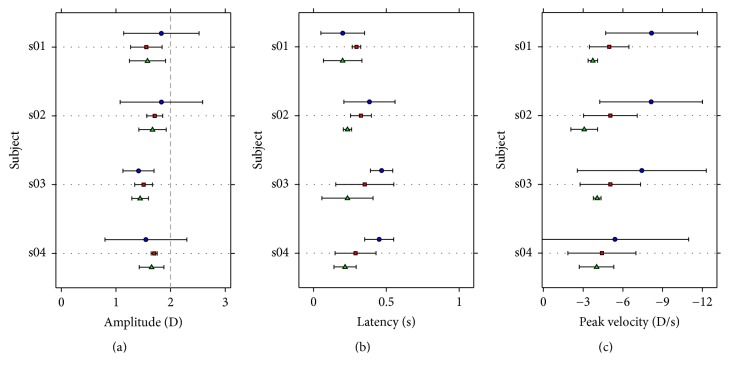
Mean obtained over the three trials for each subject of the disaccommodation parameters analyzed. (a) shows the mean amplitude of the disaccommodation response in diopters, (b) shows mean latency in seconds, and (c) shows peak velocity in diopters per second. Other details as in Figure 4.

Figure 5 shows the same results as Figure 4, but this time for disaccommodation, or in other words, when the stimulus changed its accommodation demand from 2 to 0 D.

For disaccommodation, the peak velocity is again systematically greater when the exponential fitting is used to compute it. The latency obtained with this method is greater than the one obtained with the other two methods for all but one subject. The amplitude of the responses is very similar among methods.

In the case of disaccommodation, the one-way ANOVA revealed that there were no statistically significant differences among methods only for the response amplitude (*p* = 0.900). There were statistically significant differences in the latency and the peak velocity depending on the method used to compute them (*p* = 0.017 and *p* = 0.007, resp.). Post hoc multiple pairwise comparisons revealed significant differences between latency and peak velocity calculated using the exponential fitting and those calculated when smoothing the responses (*p* = 0.013; *p* = 0.006). The same analysis shown for accommodation, but this time for disaccommodation, can be found in Table 2.

## 4. Discussion

The aim of this study was to assess if the use of different analysis strategies to characterize accommodation and disaccommodation dynamics results in differences in the values of amplitude, latency, and peak velocity of accommodation and disaccommodation responses to pulse changes in demand.

Albeit the large variability in first-order accommodation and disaccommodation dynamics, demonstrated by previous studies [15, 20], the values obtained here are in agreement with those studies [6, 7], considering the group age (between 20 and 30), the amplitude of the demand (2 D), and the starting point (0 D for accommodation and 2 D for disaccommodation). The large error bars obtained for some subjects and parameters were also expected, since there is certain variability in responses, even within the same subject. Although the sample size used in this work is small, it is thought to be enough to accomplish the goal described previously, given the fact that this goal was to look for possible differences in the parameters depending on the strategy used.

Similar values of amplitude and latency of the responses were obtained for accommodation and disaccommodation, which is in agreement with previous work [7]. The peak velocity was slightly greater in magnitude for disaccommodation, with the differences being greater when the exponential fitting was used. Greater peak velocity in disaccommodation has been also reported previously [6, 20].

Differences and disagreement found among previous studies can be due to several reasons, such as the significant interindividual variability exhibited in these kinds of step responses [15, 20]. Another reason may be the fact that the parameters characterizing accommodation and disaccommodation dynamics have been computed using different strategies, such as exponential fitting [7, 21], sigmoidal fitting [22, 23], or analyzing directly the experimental data, with or without previous smoothing [16]. The results obtained here highlight the fact that the use of a certain strategy can yield different results, mostly in peak velocity, given the fact that the derivative of the data is quite different whether a function is fitted or the experimental data is carefully smoothed. Generally, the responses analyzed in this study seemed to follow a sigmoidal function more closely than an exponential. This is likely the reason the peak velocity presents the largest variability when the exponential fitting is used.

**Figure 6:**
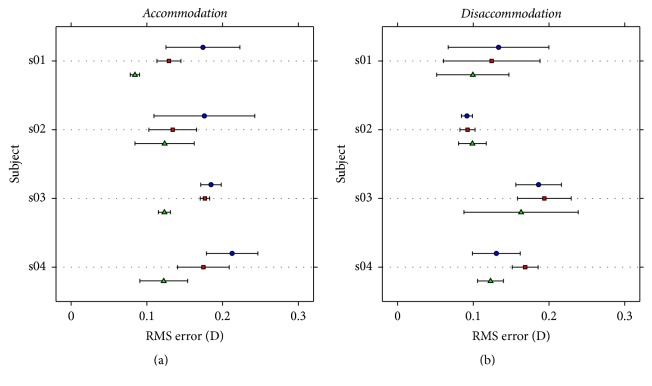
Mean root mean square (RMS) error in diopters between the fitted function and the experimental data for each subject and for the three different methodologies. (a) shows the RMS for accommodation responses, whereas (b) shows the RMS for disaccommodation responses. Other details as in Figure 4.

Due to the differences found among the different strategies, the best way to analyze these data is to carefully choose the strategy to follow for each individual response. One way could be to look at the root mean square (RMS) error obtained between the fitted function and the original data. In this regard, Figure 6 shows the mean RMS error obtained for each one of the three strategies used here and for each subject.

For accommodation, smoothing the data seems to be the closest match to the original responses, followed by the sigmoidal fitting. For disaccommodation, the mean RMS error is similar among the three strategies. The ideal scenario would be to analyze directly the experimental response; however, this also presents drawbacks. The experimental data can have a fair amount of noise and undesired fluctuations, which can make the algorithms for searching the starting or ending point of the responses behave suboptimally. Another disadvantage is the fact that, in order to analyze the velocity pattern of the responses, the first derivative of the data must be calculated. When there are fluctuations and noise in the original response, its derivative presents large amounts of noise, making it extremely hard to analyze properly and to extract parameters, such as the peak velocity. By fitting responses to smooth functions, for example, sigmoid, this problem is solved, obtaining also a smooth derivative.

## 5. Conclusions

In conclusion, amplitude, latency, and especially peak velocity values in accommodation and disaccommodation dynamics are different depending on the strategy or methodology used to compute them. This can be one of the reasons behind the disagreement among different studies in the past. When looking at the experimental responses, generally they seem to be closer to a sigmoid function; however, due to the large interindividual variability, parameters such as the RMS error should be used in order to choose the best strategy to analyze each response.

## Figures and Tables

**Table 1:** Post hoc statistical analysis of the parameters analyzed for accommodation. A bold *p* value means that statistical differences were found between the results yielded by that pair of methodologies. CI: confidence interval; amp: amplitude; lat: latency; pV: peak velocity. 1 refers to the exponential fitting; 2 refers to the sigmoid fitting; 3 refers to the smoothing.

Accommodation	Mean of the differences	Lower limit 95% CI	Upper limit 95% CI	*p* value
amp1-amp2 (D)	0.122	−0.253	0.497	0.705
amp1-amp3 (D)	0.185	−0.190	0.560	0.457
amp2-amp3 (D)	0.062	−0.313	0.437	0.913
lat1-lat2 (s)	0.112	−0.072	0.298	0.304
lat1-lat3 (s)	0.097	−0.088	0.281	0.414
lat2-lat3 (s)	−0.016	−0.201	0.168	0.974
pV1-pV2 (D/s)	1.697	−0.099	3.494	0.067
pV1-pV3 (D/s)	2.362	0.566	4.159	**0.008**
pV2-pV3 (D/s)	0.665	−1.132	2.461	0.639

**Table 2:** Post hoc* statistical *analysis of the parameters analyzed for disaccommodation. A bold *p* value means that statistical differences were found between the results yielded by that pair of methodologies. CI: confidence interval; amp: amplitude; lat: latency; pV: peak velocity. 1 refers to the exponential fitting; 2 refers to the sigmoid fitting; 3 refers to the smoothing.

Disaccommodation	Mean of the differences	Lower limit 95% CI	Upper limit 95% CI	*p* value
amp1-amp2 (D)	0.038	−0.341	0.417	0.967
amp1-amp3 (D)	0.071	−0.308	0.450	0.891
amp2-amp3 (D)	0.033	−0.346	0.412	0.975
lat1-lat2 (s)	0.060	−0.066	0.186	0.478
lat1-lat3 (s)	0.155	0.029	0.281	**0.013**
lat2-lat3 (s)	0.095	−0.032	0.221	0.172
pV1-pV2 (D/s)	−2.408	−5.018	0.201	0.075
pV1-pV3 (D/s)	−3.560	−6.170	−0.951	**0.006**
pV2-pV3 (D/s)	−1.152	−3.761	1.457	0.531
